# Supplementary material for: Common and distinct predictors of non-symbolic and symbolic ordinal number processing across the early primary school years
Source: PLoS One. 2021 Oct 21;16(10):e0258847. doi: 10.1371/journal.pone.0258847 (PMC8530342; doi:10.1371/journal.pone.0258847)
Supplement: S1 Table — Note. For all predictors, unstandardized regression coefficients are reported (standard errors in parentheses). LCI denotes the lower end of the 95% confidence interval, and UCI denotes the upper end of the 95% confidence interval. (DOCX) [file pone.0258847.s001.docx]

| **Models** | **Non-symbolic ordering** | | | | | | |  | **Symbolic ordering** | | | | |
| --- | --- | --- | --- | --- | --- | --- | --- | --- | --- | --- | --- | --- | --- |
|  | ***B (SE)*** | **LCI** | | **UCI** | | | ***p*** |  | ***B (SE)*** | **LCI** | | **UCI** | ***p*** |
| **Model 1** |  |  | | |  | |  |  |  |  |  | |  |
| Processing speed | .21 (.06) | .09 | | | .33 | | .001 |  | .35 (.08) | .19 | .51 | | < .001 |
| Model Fit | *F* = 12.01, *p* = .001, adj. *R^2^* = .07 | | | | | | |  | *F* = 18.16, *p* < .001, adj. *R^2^* = .10 | | | | |
| **Model 2** |  |  | | |  | |  |  |  |  |  | |  |
| Processing speed | .19 (.06) | .07 | | | .31 | | .003 |  | .31 (.08) | .14 | .46 | | < .001 |
| Verbal storage | .08 (.12) | -.16 | | | .30 | | .508 |  | .12 (.15) | -.18 | .42 | | .437 |
| Visuo-spatial storage | .19 (.13) | -.06 | | | .44 | | .129 |  | .41 (.17) | .08 | .74 | | .015 |
| Model Fit | *F* = 5.06, *p* = .002, adj. *R^2^* = .07 | | | | | | |  | *F* = 8.68, *p* < .001, adj. *R^2^* = .13 | | | | |
| **Model 3** |  |  | | |  | |  |  |  |  |  | |  |
| Processing speed | .17 (.06) | .05 | | | .29 | | .005 |  | .27 (.08) | .12 | .43 | | < .001 |
| Verbal storage | .03 (.11) | -.19 | | | .26 | | .77 |  | .07 (.15) | -.23 | .35 | | .669 |
| Visuo-spatial storage | -.01 (.13) | -.27 | | | .25 | | .92 |  | .11 (.17) | -.23 | .44 | | .530 |
| Verbal manipulation | -.06 (.15) | -.35 | | | .24 | | .72 |  | -.15 (.19) | -.53 | .24 | | .451 |
| Visuo-spatial manipulation | .38 (.09) | .20 | | | .57 | | < .001 |  | .58 (.12) | .35 | .82 | | < .001 |
| Model Fit | *F* = 6.83, *p* < .001, adj. *R^2^* = .16 | | | | | | |  | *F* = 10.70, *p* < .001, adj. *R^2^* = .24 | | | | |
| **Model 4** |  |  | | |  |  | |  |  |  |  | |  |
| Processing speed | .07 (.09) | -.06 | | | .20 | .261 | |  | .13 (.08) | -.04 | .29 | | .125 |
| Verbal storage | .02 (.11) | -.19 | | | .23 | .833 | |  | .06 (.14) | -.21 | .33 | | .673 |
| Visuo-spatial storage | -.04 (.13) | -.29 | | | .21 | .741 | |  | .06 (.16) | -.26 | .37 | | .721 |
| Verbal manipulation | -.05 (.14) | -.33 | | | .23 | .713 | |  | -.14 (.18) | -.50 | .21 | | .428 |
| Visuo-spatial manipulation | .26 (.09) | .08 | | | .44 | .004 | |  | .41 (.12) | .19 | .64 | | < .001 |
| Non-symbolic comparison | 1.98 (.50) | 1.00 | | | 2.96 | < .001 | |  | 2.44 (.64) | 1.18 | 3.70 | | < .001 |
| Symbolic comparison | -.06 (.55) | -1.14 | | | 1.02 | .914 | |  | .25 (.70) | -1.14 | 1.65 | | .719 |
| Model Fit | *F* = 9.14, *p* < .001, adj. *R^2^* = .27 | | | | | | |  | *F* = 12.61, *p* < .001, adj. *R^2^* = .34 | | | | |
| **Model 5** |  | |  | |  |  | |  |  |  |  | |  |
| Processing speed | .07 (.07) | | -.07 | | .20 | .329 | |  | .07 (.09) | -.09 | .24 | | .384 |
| Verbal storage | .02 (.11) | | -.19 | | .23 | .848 | |  | .04 (.14) | -.22 | .31 | | .754 |
| Visuo-spatial storage | -.04 (.13) | | -.29 | | .21 | .745 | |  | .06 (.16) | -.25 | .37 | | .698 |
| Verbal manipulation | -.05 (.14) | | -.33 | | .23 | .742 | |  | -.10 (.17) | -.45 | .25 | | .564 |
| Visuo-spatial manipulation | .26 (.09) | | .08 | | .44 | .005 | |  | .40 (.11) | .17 | .62 | | .001 |
| Non-symbolic comparison | 1.98 (.50) | | .99 | | 2.96 | < .001 | |  | 2.38 (.63) | 1.13 | 3.62 | | < .001 |
| Symbolic comparison | -.07 (.55) | | -1.16 | | 1.02 | .899 | |  | .17 (.70) | -1.20 | 1.54 | | .806 |
| Counting | .53 (1.44) | | -2.31 | | 3.38 | .712 | |  | 4.20 (1.82) | .61 | 7.79 | | .022 |
| Model Fit | *F* = 7.97, *p* < .001, adj. *R^2^* = .26 | | | | | | |  | *F* = 12.03, *p* < .001, adj. *R^2^* = .36 | | | | |
