# Supplementary material for: Common and distinct predictors of non-symbolic and symbolic ordinal number processing across the early primary school years
Source: PLoS One. 2021 Oct 21;16(10):e0258847. doi: 10.1371/journal.pone.0258847 (PMC8530342; doi:10.1371/journal.pone.0258847)
Supplement: S2 Table — Note. For all predictors, unstandardized regression coefficients are reported (standard errors in parentheses). LCI denotes the lower end of the 95% confidence interval, and UCI denotes the upper end of the 95% confidence interval. (DOCX) [file pone.0258847.s002.docx]

| **Models** | **Non-symbolic ordering** | | | | |  | **Symbolic ordering** | | | |
| --- | --- | --- | --- | --- | --- | --- | --- | --- | --- | --- |
|  | ***B (SE)*** | **LCI** | **UCI** | | ***p*** |  | ***B (SE)*** | **LCI** | **UCI** | ***p*** |
| Processing speed | .01 (.01) | -.02 | | .04 | .371 |  | .01 (.02) | -.02 | .04 | .442 |
| Verbal storage | .01 (.02) | -.03 | | .06 | .577 |  | -.01 (.02) | -.06 | .04 | .821 |
| Visuo-spatial storage | .03 (.03) | -.02 | | .09 | .227 |  | .02 (.03) | -.04 | .07 | .480 |
| Verbal manipulation | -.01 (.03) | -.07 | | .05 | .840 |  | .05 (.03) | -.01 | .11 | .117 |
| Visuo-spatial manipulation | .04 (.02) | .00 | | .08 | .041 |  | .04 (.02) | .00 | .08 | .045 |
| Non-symbolic comparison | -.03 (.11) | -.24 | | .18 | .777 |  | -.30 (.11) | -.52 | -.09 | .006 |
| Symbolic comparison | .25 (.12) | .01 | | .48 | .039 |  | .29 (.12) | .05 | .53 | .019 |
| Counting | .06 (.31) | -.55 | | .67 | .839 |  | .15 (.32) | -.48 | .77 | .642 |
| Model Fit | *F* = 4.42, *p* < .001, adj. *R^2^* = .15 | | | | |  | *F* = 3.29, *p* = .002, adj. *R^2^* = .11 | | | |
