# Supplementary material for: Common and distinct predictors of non-symbolic and symbolic ordinal number processing across the early primary school years
Source: PLoS One. 2021 Oct 21;16(10):e0258847. doi: 10.1371/journal.pone.0258847 (PMC8530342; doi:10.1371/journal.pone.0258847)
Supplement: S3 Table — Note. For all predictors, unstandardized regression coefficients are reported (standard errors in parentheses). LCI denotes the lower end of the 95% confidence interval, and UCI denotes the upper end of the 95% confidence interval. (DOCX) [file pone.0258847.s003.docx]

| **Models** | **Non-symbolic ordering** | | | | |  | **Symbolic ordering** | | | |
| --- | --- | --- | --- | --- | --- | --- | --- | --- | --- | --- |
|  | ***B (SE)*** | **LCI** | **UCI** | | ***p*** |  | ***B (SE)*** | **LCI** | **UCI** | ***p*** |
| Processing speed | .02 (.02) | -.02 | | .05 | .327 |  | .02 (.02) | -.01 | .06 | .123 |
| Verbal storage | -.02 (.02) | -.07 | | .03 | .508 |  | .01 (.03) | -.04 | .06 | .777 |
| Visuo-spatial storage | .04 (.03) | -.02 | | .10 | .167 |  | .02 (.03) | -.04 | .08 | .505 |
| Verbal manipulation | .02 (.03) | -.05 | | .08 | .634 |  | .01 (.03) | -.06 | .07 | .792 |
| Visuo-spatial manipulation | -.02 (.02) | -.06 | | .02 | .320 |  | .01 (.02) | -.04 | .05 | .686 |
| Non-symbolic comparison | .12 (.11) | -.11 | | .34 | .317 |  | -.05 (.12) | -.28 | .18 | .645 |
| Symbolic comparison | .09 (.13) | -.16 | | .34 | .459 |  | .12 (.13) | -.13 | .38 | .331 |
| Counting | -.25 (.33) | -.90 | | .41 | .456 |  | -.39 (.33) | -1.05 | .27 | .245 |
| Model Fit | *F* = 1.60, *p* = .131, adj. *R^2^* = .03 | | | | |  | *F* = 1.19, *p* = .310, adj. *R^2^* = .01 | | | |
