# Supplementary material for: Common and distinct predictors of non-symbolic and symbolic ordinal number processing across the early primary school years
Source: PLoS One. 2021 Oct 21;16(10):e0258847. doi: 10.1371/journal.pone.0258847 (PMC8530342; doi:10.1371/journal.pone.0258847)
Supplement: S4 Table — Note. For all predictors, unstandardized regression coefficients are reported (standard errors in parentheses). r refers to the correlation between a given predictor and ordering processing. (DOCX) [file pone.0258847.s004.docx]

| **Models** | ***B (SE)*** | **95% CI** | | ***p*** | ***r*** |
| --- | --- | --- | --- | --- | --- |
|  |  | **Lower** | **Upper** |  |  |
| **Model 1: Processing speed, working memory and symbolic number skills** |  |  |  |  |  |
| Processing speed | .01 (.02) | -.02 | .04 | .451 | .17 |
| Verbal storage | -.01 (.02) | -.06 | .04 | .632 | .04 |
| Visuo-spatial storage | .02 (.03) | -.03 | .08 | .395 | .21 |
| Verbal manipulation | .05 (.03) | -.01 | .11 | .121 | .19 |
| Visuo-spatial manipulation | .03 (.02) | -.01 | .07 | .120 | .25 |
| Symbolic comparison | .08 (.10) | -.11 | .28 | .395 | .22 |
| Counting | .11 (.32) | -.53 | .75 | .732 | .10 |
| Model Fit | .01 (.02) | | | | |
| **Model 2: With non-symbolic comparison** |  |  |  |  |  |
| Processing speed | .01 (.02) | -.02 | .04 | .442 | .17 |
| Verbal storage | -.01 (.02) | -.05 | .04 | .821 | .04 |
| Visuo-spatial storage | .02 (.03) | -.04 | .07 | .480 | .21 |
| Verbal manipulation | .05 (.03) | -.01 | .11 | .117 | .19 |
| Visuo-spatial manipulation | .04 (.02) | .00 | .08 | .045 | .25 |
| Symbolic comparison | .29 (.12) | .05 | .53 | .019 | .22 |
| Counting | .15 (.32) | .48 | .77 | .642 | .10 |
| Non-symbolic comparison | -.30 (.11) | -.52 | -.09 | .006 | .03 |
| Model Fit | *F* = 2.93, *p* = .002, adjusted *R^2^* = .11 | | | | |
